# Parental origin of monosomic chromosomes in near-haploid acute lymphoblastic leukemia

**DOI:** 10.1038/s41408-020-0317-2

**Published:** 2020-05-05

**Authors:** Kristina B. Lundin-Ström, Kristoffer Ström, Andrea Biloglav, Gisela Barbany, Mikael Behrendtz, Anders Castor, Linda Olsson-Arvidsson, Bertil Johansson

**Affiliations:** 1 Division of Clinical Genetics, Department of Laboratory Medicine, Lund University, Lund, Sweden; 2 Lund University Diabetes Centre, Department of Clinical Sciences, Lund University, Malmö, Sweden; 3 Department of Molecular Medicine and Surgery, Center for Molecular Medicine, Karolinska Institutet, Stockholm, Sweden; 4 Department of Pediatrics, Linköping University Hospital, Linköping, Sweden; 5 Department of Pediatrics, Skåne University Hospital, Lund, Sweden; 6 Department of Clinical Genetics and Pathology, Division of Laboratory Medicine, Lund, Sweden

**Keywords:** Genetics research, Cancer epigenetics

Dear Editor,

Near-haploid acute lymphoblastic leukemia (NH ALL), defined as having a modal chromosome number between 25 and 29, is genetically characterized by the presence of (i) numerous monosomies and only a few disomies, the latter mainly involving the sex chromosomes and the autosomes 10, 14, 18, and, always, 21; (ii) subclones with 50–58 chromosomes, representing karyotypically exact duplicates of the NH clones that result in uniparental isodisomies (UPIDs) of all originally monosomic chromosomes; (iii) mutations/deletions targeting proteins involved in RTK/RAS signaling, histone modification, and transcription; in contrast, cytogenetically identifiable structural changes are rare with no recurrent fusion genes having been reported^1,2^. Clinical characteristics include the presence of small and large lymphoblasts representing the NH and the doubled-up subclones, respectively, a B-cell precursor immunophenotype, young age, and an achievable but unfortunately often short remission. Thus, the prognosis is generally poor.

How the near-haploid pattern arises has not been clarified—an initial multipolar mitosis or partial mitotic, i.e., somatic pairing of homologous chromosomes has been suggested but without any experimental evidence supporting either mechanism^1,3^. Also, the pathogenetic consequences of near-haploidy are unknown; both loss of tumor-suppressor genes and global gene-dosage effects have been proposed. An issue that has never been addressed is how NH ALL cells not only can survive but also apparently “prosper” in vivo, despite the massive loss of genes. This question is relevant not least considering that a relatively large proportion of human genes displays a monoallelic expression pattern due to, for example, imprinting^4^. Because numerous genes in NH ALL cells hence are expected to be unexpressed, we hypothesized that there could be a bias as to which parentally imprinted chromosomes are monosomic—the “active” or the “inactive” ones?

In this study, the first to investigate the parental origin of monosomies in NH ALL, we analyzed the methylation patterns of imprinted gene loci in order to ascertain whether the maternal or paternal copies are preferentially retained in this ALL subtype. Chromosomes 6 (*PLAGL1* gene), 7 (*PEG10*), 11 (*H19*), 14 (*MEG3*), and 15 (*SNRPN*) were selected for analysis because UPIDs of these chromosomes lead to well-known imprinting disorders for which validated methylation analyses are available: (i) paternal UPID6 results in transient neonatal diabetes mellitus; (ii) maternal UPID7 leads to Russell–Silver syndrome as does duplication of the maternal copy or loss of methylation of the paternal copy of an imprinting center at 11p15; (iii) copy number neutral loss of heterozygosity (CN-LOH) of the paternal 11p15 is one of several mechanisms underlying Beckwith–Wiedemann syndrome; (iv) maternal and paternal UPID14 result in the Temple and Kagami–Ogata syndromes, respectively; (v) maternal and paternal UPIDs of chromosome 15 are associated with Prader–Willi and Angelman syndromes, respectively^5^. Thus, maternal and paternal monosomies/UPIDs of these five chromosomes can be distinguished by analyzing the methylation patterns of the imprinted *PLAGL1*, *PEG10*, *H19*, *MEG3*, and *SNRPN* genes, respectively.

The NH ALL cases analyzed comprised seven patient samples and the two near-haploid cell lines NALM-16 and MHH-CALL-2, obtained from DSMZ GmbH (ACC-680 and ACC-341, respectively, both mycoplasma negative). The limited number of cases reflects both the rarity of NH ALL and restricted availability of DNA, but is sufficient to achieve significance if a particular parental chromosome is retained in all or eight of the nine cases (*P* < 0.05, two-tailed exact binomial calculation). All had monosomies or, in the case of duplicated clones, UPIDs of chromosomes 6, 7, 11, and 15, and three of them also had monosomy/UPID of chromosome 14 (Table 1).

**Table 1:** Parental origins of monosomic chromosomes in near-haploid acute lymphoblastic leukemia.

Case no.	Monosomies/UPIDs	Parental copies present based on the methylation status of the retained imprinted genes				
		Chr 6 (*PLAGL1*)	Chr 7 (*PEG10*)	Chr 11 (*H19*)	Chr 14 (*MEG3*)	Chr 15 (*SNRPN*)
1	6, 7, 11, 15	Pat (U)	Pat (U)	Mat (U)	Mat/pat (U/M)	Mat (M)
2	6, 7, 11, 15	Pat (U)	Pat (U)	Mat (U)	Mat/pat (U/M)	Pat (U)^a^
2 (relapse)	6, 7, 11, 15	Pat (U)	Pat (U)	Mat (U)	Mat/pat (U/M)	Pat (U)^a^
3	6, 7, 11, 14, 15	Pat (U)	Pat (U)	Mat (U)	Pat (M)	Pat (U)^a^
4	6, 7, 11, 15	Mat (M)	Mat (M)	Pat (M)	Mat/pat (U/M)	Mat (M)^a^
5	6, 7, 11, 15	Mat (M)	Mat (M)	Mat (U)	Mat/pat (U/M)	Pat (U)^a^
6	6, 7, 11, 14, 15	Mat (M)	Mat (M)	Mat (U)	Pat (M)	Mat (M)^a^
7	6, 7, 11, 15	Mat (M)	Pat (U)	Mat (U)	Mat/pat (U/M)	Pat (U)^a^
MHH-CALL-2	6, 7, 11, 14, 15	Mat (M)	Mat (M)	Mat (U)	Mat (U)	Pat (U)
NALM-16	6, 7, 11, 15	Pat (U)	Mat (M)	Mat (U)	Mat/pat (U/M)	Mat (M)
		*P* = 1.0	*P* = 1.0	*P* = 0.039	*P* = 1.0	*P* = 1.0

*M* methylated, *Mat* maternal, *Pat* paternal, *U* unmethylated, *UPIDs* uniparental isodisomies.

^a^A weak signal of the methylated (maternal) gene copy (cases 2, 3, 5, and 7) and of the unmethylated (paternal) gene copy (cases 4 and 6) was also present, most likely due to a combination of a highly sensitive PCR analysis and an admixture of non-leukemic cells in the samples.

Genomic DNA from the leukemic samples, a healthy subject, and a universally methylated control (Methylated Human DNA Standard, Zymo Research, CA, USA) was bisulfite converted, and *PLAGL1*, *PEG10*, and *H19* were analyzed with bisulfite sequencing, whereas methylation-specific PCR was used for *MEG3* and *SNRPN* (Supplementary Material and Supplementary Table 1). All genes were analyzed twice. The study was approved by the Research Ethics Committee of Lund University, and informed consent for the analyses was obtained according to the Declaration of Helsinki.

Monosomies/UPIDs of chromosomes 6, 7, 14, and 15 did not display any significant parent-of-origin skewness, i.e., the methylated and unmethylated imprinted loci on these chromosomes were variably retained in NH ALL (Table 1). In contrast, the *H19* locus on chromosome 11 was unmethylated, i.e., of maternal origin, in eight of the nine cases (*P* = 0.039, uncorrected for multiple testing, Table 1). Due to the possible skewness of the parental origin of chromosome 11, its impact on the expression of imprinted genes on 11p15 was ascertained by quantitative reverse transcription PCR (RT-qPCR) analyses of the paternally imprinted *CDKN1C*, *H19*, *HOTS*, *KCNQ1*, *PHLDA2*, and *SLC22A18* genes and the maternally imprinted *IGF2* and *KCNQ1OT1* genes (Supplementary Table 2). RNA of sufficient amount/quality was avaible from three NH ALLs: case 2 (diagnostic and relapse samples), MHH-CALL-2, and NALM-16 (Supplementary Table 3). In addition, RNA from two cell lines with disomy 11 was included as control to validate the RT-qPCR assay used: the acute monocytic leukemia cell line THP-1 (ACC-16, DSMZ) and the human mammary endothelial cell line HMEC-1 (ATCC^®^ CRL-3243), both mycoplasma negative. The expression levels of the *H19*, *KCNQ1OT1*, *PHLDA2*, and *SLC22A18* genes were relatively low compared with the levels seen in the monocytic THP-1 and/or the endothelial HMEC-1 cell lines. No measurable expression of *IGF2* was found in the NH ALL cases or in the THP-1 and HMEC-1 cell lines (Supplementary Table 3).

Although there have been many studies on acquired imprinting defects in human malignancies, they have mainly focused on abnormal gene expression patterns linked to other epigenetic features, such as loss of imprinting, resulting in activation of imprinted alleles through DNA demethylation, or silencing of active alleles through DNA methylation, rather than on changes at the DNA level^6^. On the other hand, investigations of acquired UPIDs/CN-LOH regions, which occur at the genomic level, have rarely analyzed their impact on expression of imprinted loci or on their parental origins. Instead, in most instances, the emphasis has been on identifying homozygously mutated genes in regions with CN-LOH^7–9^. In fact, we know of only a handful of studies focusing on which maternal or paternal homologs are involved in segments with acquired CN-LOH. Parental bias has been described for CN-LOH of 14q in myeloproliferative neoplasms and myelodysplastic syndromes, in which the imprinted *DLK1*/*MEG3* domain displayed increased methylation, consistent with a paternal origin of this CN-LOH; in the same study, no such parental bias was seen for CN-LOH of 22q^10^. Furthermore, acquired CN-LOH of the 11p15 region is a common feature in adrenocortical tumors, and methylation analyses of *IGF2* and *H19* in such tumors have revealed that the duplicated allele is of paternal origin in the vast majority of cases^11^. In contrast, no parental skewness for CN-LOH of 11p15 has been observed in acute myeloid leukemias or inflammatory leiomyosarcomas^12,13^.

Whether the parental origin of acquired chromosome changes is pathogenetically important in ALL is unknown. It has been reported that 9p deletions in BCP ALL preferentially involve the maternal homolog of chromosome 9; however, several exceptions have been reported and, furthermore, these studies were based on “old-fashioned” methods that easily could have missed smaller deletions^14^. With regard to whole-chromosome losses and gains in NH ALL and high hyperdiploid (HeH) cases, respectively, Haas suggested more than 20 years ago that imprinting could play a role in these subtypes, although this hypothesis could not be confirmed for HeH, where the parental origin of the additional chromosomes did not show any bias^14,15^.

The present study is the first to investigate the parental origin of monosomic chromosomes in NH ALL. By using the methylation status of imprinted gene loci to distinguish between maternal and paternal homologs of chromosomes 6, 7, 11, 14, and 15, we here show that there was no indication for a parent-of-origin bias for most of them (Table 1). The exception was monosomy 11, in which the *H19* locus was unmethylated, i.e., of maternal origin, in eight of the nine cases. However, this skewed distribution was not statistically significant if corrected for multiple testing, and could be fortuitous due to the limited number of cases included in the analyses. Furthermore, even if there is a preferential retainment of the maternal chromosome 11 in NH ALL, its pathogenetic impact is clearly not due to aberrant expression of the genes in the imprinting centers at 11p15—the present RT-qPCR analyses revealed that they were either unexpressed or only expressed at low levels (Supplementary Table 3). Taken together, our results strongly suggest that loss of imprinting caused by the monosomies in NH ALL does not play a major pathogenetic role, and that the maternal and paternal chromosome homologs are randomly lost in this leukemia type. However, retainment of the maternal chromosome 11 or, alternatively, loss of the paternal homolog, may provide a selective advantage in NH ALL, although this needs to be confirmed in an independent series of cases.

## Supplementary information

Supplemental material
