# Supplementary material for: Parental origin of monosomic chromosomes in near-haploid acute lymphoblastic leukemia
Source: Blood Cancer J. 2020 May 5;10(5):51. doi: 10.1038/s41408-020-0317-2 (PMC7200744; doi:10.1038/s41408-020-0317-2)
Supplement: Supplementary file 1 — Supplemental material [file 41408_2020_317_MOESM1_ESM.docx]

**Supplementary material**

**Methods**

**Bisulfite sequencing**

Bisulfite-converted DNA was PCR-amplified using the ZymoTaq Premix (Zymo Research, CA, USA). The gel-purified products (QIAquick gel extraction kit, Qiagen, Hilden, Germany) were directly sequenced using the BigDye Terminator v1.1 Cycle Sequencing Kit according to the manufacturer’s guidelines (Applied Biosystems, CA, USA). The products were run on an ABI 3130 Genetic Analyzer (Applied Biosystems) and the results were visualized in the freeware Chromas.exe for Windows (Technelysium Pty Ltd, South Brisbane, Australia). Primer sequences are given in Supplementary Table 1.

**Methylation-specific PCR**

Bisulfite-converted DNA was amplified in two separate PCR-reactions (ZymoTaq Premix) with different primer pairs for the unmethylated and methylated alleles. The PCR products were run on 1% agarose gels and visually inspected (present/absent compared with the controls). Primer sequences are given in Supplementary Table 1.

**RT-qPCR**

Reverse transcription was performed on 1.0 µg RNA with the QuantiTect reverse transcription kit (Qiagen) and RT-qPCR was carried out using the ViiA 7 Real-Time PCR System (384-well setting with each well containing 8 ng of cDNA in 10 µl reactions) and gene-specific TaqMan assays (Supplementary Table 2) using the TaqMan Gene Expression Master Mix (Thermo Fisher Scientific, Waltham, MA, USA) according to the manufacturer’s recommendations. The samples were analyzed in triplicates and the maximum accepted standard deviation between Ct values was 0.1 cycles. Four endogenous control genes (*GAPDH*, *HPRT1*, *POLR2A*, and *PPIA*) were included on the same plate. An internal calibrator sample was added in each RT-qPCR to correct for batch to batch variations. The expression levels were calculated and normalized by geometric averaging of the endogenous controls as previously described^1^.

**Reference**

1. Vandesompele J, et al. Accurate normalization of real-time quantitative RT-PCR data by geometric averaging of multiple internal control genes. Genome Biol. 2002;3(7):RESEARCH0034.

**Supplementary Table 1** Primers and methods used to analyze the methylation status of *PLAGL1*, *PEG10*, *H19*, *MEG3*, and *SNRPN*

| Primer | Sequence (5’→3’) | Method | Product | Reference |
| --- | --- | --- | --- | --- |
|  |  |  | size (bp) |  |
| PLAGL1-F | GTGTGGGTGTYGTTTAGTTTTTTT | BSS | 224 | 1 |
| PLAGL1-R | AACTAAATAACAAATAACAAATACC | BSS | 224 | 1 |
| PEG10-F | AGAAACCTGACTGCGCCCTGAGGAGAACA | BSS | 155 | 2 |
| PEG10-R | TTAAGGTGTGGGATTTTATTTTTTTTGT | BSS | 155 | 2 |
| **H19-F1** | **GAGTTTGGGGGTTTTTGTATAGTAT** | **BSS** | **338** | **3** |
| **H19-R1** | **CTTAAATCCCAAACCAATAACACTA** | **BSS** | **338** | **3** |
| **H19-F2** | TATGGGTATTTTTGGAGGTTTTTTT | BSS | 316 | 4 |
| H19-R2 | AACTTAAATCCCAAACCATAACACT | BSS | 316 | 4 |
| MEG3-FM | GTTAGTAATCGGGTTTGTCGGC | MSP | 160 | 5 |
| MEG3-RM | AATCATAACTCCGAACACCCGCG | MSP | 160 | 5 |
| MEG3-FU | GAGGATGGTTAGTTATTGGGGT | MSP | 120 | 5 |
| MEG3-RU | CCACCATAACCAACACCCTATAATCACA | MSP | 120 | 5 |
| SNRPN-FM | TAAATAAGTACGTTTGCGCGGTC | MSP | 174 | 5 |
| SNRPN-RM | AACCTTACCCGCTCCATCGCG | MSP | 174 | 5 |
| SNRPN-FU | GTAGGTTGGTGTGTATGTTTAGGT | MSP | 100 | 5 |
| SNRPN-RU | ACATCAAACATCTCCAACAACCA | MSP | 100 | 5 |

BSS, bisulfite sequencing; F, forward primer; FM, forward primer for the methylated allele; FU, forward primer for the unmethylated allele; MSP, methylation specific PCR; R, reverse primer; RM, reverse primer for the methylated allele; RU, reverse primer for the unmethylated allele.

**References**

1. Bliek J, et al. Hypomethylation at multiple maternally methylated imprinted regions including *PLAGL1* and *GNAS* loci in Beckwith-Wiedemann syndrome. *Eur J Hum Genet* 2009; 17: 611-619.

2. Nakamura A, et al. A case of paternal uniparental isodisomy for chromosome 7 associated with overgrowth. *J Med Genet* 2018; 55: 567-570.

3. C**ui H, et al. Loss of imprinting in colorectal cancer linked to hypomethylation of *H19* and *IGF2*. *Cancer Res* 2002; 62: 6442-6446.**

**4. O**llikainen M, et al. DNA methylation analysis of multiple tissues from newborn twins reveals both genetic and intrauterine components to variation in the human neonatal epigenome. *Hum Mol Genet* 2010; 19: 4176-4188.

5. Benetatos L, et al. CpG methylation analysis of the MEG3 and SNRPN imprinted genes in acute myeloid leukemia and myelodysplastic syndromes. *Leuk Res* 2010; 34:148-153.

**Supplementary Table 2** TaqMan assays used for RT-qPCR expression analyses of the imprinted genes and the control genes

| Imprinted genes | Imprinted allele | Catalogue No. | Fluorescent label |
| --- | --- | --- | --- |
| *CDKN1C* | Paternal | Hs00175938_m1 | FAM-MGB |
| *H19* | Paternal | Hs00399294_g1 | FAM-MGB |
| *HOTS* | Paternal | Hs04935240_s1 | FAM-MGB |
| *IGF2* | Maternal | Hs04188276_m1 | FAM-MGB |
| *IGF2* | Maternal | Hs04971693_m1 | FAM-MGB |
| *KCNQ1* | **Paternal** | Hs00923522_m1 | **FAM-MGB** |
| *KCNQ1OT1* | **Maternal** | Hs04991170_s1 | **FAM-MGB** |
| ***PHLDA2*** | Paternal | **Hs00169368_m1** | FAM-MGB |
| ***SLC22A18*** | Paternal | **Hs00945415_m1** | FAM-MGB |
|  |  |  |  |
| Control genes |  |  |  |
| *GAPDH* | NA | Hs02758991_g1 | FAM-MGB |
| *HPRT1* | NA | 4326321E | VIC-MGB |
| *POLR2A* | NA | Hs00172187_m1 | FAM-MGB |
| *PPIA* (*CYCA*) | NA | 4326316E | VIC-MGB |

All assays were obatined from Thermo Fisher Scientific (Waltham, MA, USA).

FAM, fluorescein amidites; MGB, minor groove binder; NA, not applicable; VIC, Victoria (*Aequorea victoria*).

**Supplementary Table 3** Relative expression levels of the *CDKN1C*, *H19*, *HOTS*, *IGF2*, *KCNQ1*, *KCNQ1OT1*, *PHLDA2*, and ***SLC22A18* genes in relation to four endogeneous control genes (***GAPDH*, *HPRT1*, *POLR2A*, and *PPIA*)

| Samples | Imprinted genes in 11p15 | | | | | | | |
| --- | --- | --- | --- | --- | --- | --- | --- | --- |
| analyzed | *CDKN1C* | *H19* | *HOTS* | *IGF2* | *KCNQ1* | *KCNQ1OT1* | ***PHLDA2*** | ***SLC22A18*** |
| Case 2 (diagnosis) | 0.637 | 0.070 | 0.250 | - | 0.836 | 0.401 | 0.082 | 0.220 |
| Case 2 (relapse) | 0.679 | 0.081 | 0.270 | - | 0.424 | 0.146 | 0.201 | 0.151 |
| MHH CALL-2 | 1.100 | 0.015 | 0.090 | - | 1.100 | 0.137 | 0.211 | 0.424 |
| NALM-16 | 0.393 | 0.057 | 0.123 | - | 0.001 | - | 0.116 | 1.200 |
| THP1 | **0.711** | **0.002** | - | - | **1.210** | 2.242 | 2.242 | 1.611 |
| HMEC | **0.742** | **2.323** | **na** | **-** | **0.016** | **na** | **na** | **na** |

na, not analyzed; -, no measurable expression.
